# Comparative Evaluation of Quality Attributes of the Dried Cherry Blossom Subjected to Different Drying Techniques

**DOI:** 10.3390/foods13010104

**Published:** 2023-12-28

**Authors:** Kui Suo, Yabin Feng, Yang Zhang, Zhenfeng Yang, Cunshan Zhou, Wei Chen, Liyu Shi, Chunfeng Yan

**Affiliations:** 1 College of Biological and Environmental Sciences, Zhejiang Wanli University, Ningbo 315100, Chinayangzf@zwu.edu.cn (Z.Y.);; 2 School of Food and Biological Engineering, Jiangsu University, Zhenjiang 212013, China; 3 Haishu Agricultural Extension Center of Zhejiang, Ningbo 315100, China

**Keywords:** cherry blossoms, drying techniques, tea infusions, bioactive compounds, antioxidant activity, α-glucosidase activity, sensory evaluation

## Abstract

Choosing an appropriate drying method is crucial for producing dried cherry blossoms with desirable quality. This study is designed to assess the effects of seven different drying methods—hot-air drying (HAD), infrared hot-air drying (IHAD), catalytic infrared drying (CID), relative humidity drying (RHD), pulsed vacuum drying (PVD), microwave vacuum drying (MVD), and vacuum freeze drying (VFD)—on drying time and various attributes of cherry blossoms, such as appearance, bioactive compounds, antioxidant activity, α-glucosidase activity, and sensory properties. Our findings revealed that MVD recorded the shortest drying time, followed by PVD, CID, IHAD, RHD, HAD, and VFD. In qualities, VFD-dried petals exhibited superior appearance, bioactive compounds, antioxidant activity, and α-glucosidase inhibitory capability; MVD-dried petals were a close second. Furthermore, the quality of tea infusions prepared from the dried petals was found to be significantly correlated with the quality of the dried petals themselves. Regarding sensory attributes, VFD-dried petals produced tea infusions most similar in flavor and taste to those made with fresh petals and received the highest sensory evaluation scores, followed by MVD, PVD, RHD, CID, IHAD, and HAD. These results could offer a scientific foundation for the mass production of high-quality dried cherry blossoms in the future.

## 1. Introduction

The cherry blossom (*Cerasus serrulate ‘Sekiyama’*) is renowned for its aesthetic appeal and multifaceted health-promoting properties [1,2]. Notably, in 2022, the cherry blossom was formally recognized as a food raw material in China, thereby creating significant economic opportunities within the food industry. However, the brief and seasonal harvest window poses challenges to efficient distribution and consumption, thus limiting its economic utility [3]. Adopting an effective processing method is therefore essential to extend the shelf life of cherry blossoms, mitigate spoilage, and facilitate commercialization. 

Previous studies have shown that drying processes can eliminate up to 90% of the moisture content in food items, thereby inhibiting microbial spoilage, reducing water-mediated degradation reactions, and lowering transportation costs [4,5,6]. Current drying techniques for food products are broadly classified into two categories: thermal and nonthermal techniques. Thermal techniques encompass hot air, relative humidity, microwave, vacuum, and infrared drying. In contrast, vacuum freeze drying is categorized as a nonthermal technique [7,8].

Choosing appropriate drying techniques significantly influences the physicochemical and sensory quality of processed food products [9,10]. For instance, Juhari et al. [11] found that freeze-drying was superior to thermal techniques like oven-drying, vacuum-drying, and sun-drying in preserving the cell structure of roselle, rendering it most comparable to fresh samples. Li et al. [12] reported that chrysanthemums processed via temperature–humidity-controlled hot-air drying retained higher water-holding capacity and chlorogenic acid content than those subjected to infrared-assisted hot-air drying and hot-air drying. Cheng et al. [13] demonstrated that, compared to room temperature, heat pump, and freeze-drying techniques, microwave vacuum-drying was the most efficient technique for preserving the phenolic compounds and bioactivities of green coffee beans. Given these observations, the selection of an optimal drying technique is paramount for achieving the desired end-product quality. 

Furthermore, the application of drying techniques offers a novel consumption modality for edible flowers, specifically through the creation of tea infusions using dried petals. Wu et al. [14] discovered that drying techniques are well-suited for the production of high-quality tea flower beverages, based on sensory evaluation, tea polyphenols content, amino acid content, and other indexes. Yigit et al. [15] noted that the total phenolic content, total monomeric anthocyanin content, and antioxidant capacity of a hot-air-dried lilac flower infusion (brewing 10 min) were better than those of the shade-dried sample. Zheng et al. [16] mentioned that the total polyphenols and total flavonoids of loquat flower tea prepared by microwave drying were higher than those prepared by hot-air drying, and its aroma was stronger. Nonetheless, the existing literature lacks a comprehensive and systematic investigation into the impact of various drying techniques on multiple quality attributes of cherry-blossom products, including dried petals and tea infusions. Specifically, there is a need to evaluate how these techniques impact drying time, appearance, bioactive compounds, antioxidant activities, and α-glucosidase inhibitory ability, as well as sensory attributes.

Accordingly, this study aims to fill this research gap by examining the efficacy of seven drying techniques—hot-air drying (HAD), infrared hot-air drying (IHAD), catalytic infrared drying (CID), relative humidity drying (RHD), pulsed vacuum drying (PVD), microwave vacuum drying (MVD), and vacuum freeze drying (VFD)—on drying time and multiple quality attributes of dried cherry blossoms. These attributes include appearance, bioactive compound content (phenolics, flavonoids, and anthocyanins), and antioxidant activities (DPPH and ABTS radical-scavenging activity), as well as the α-glucosidase inhibitory ability of dried cherry blossoms. Concurrently, we will assess the quality attributes—comprising bioactive compounds, antioxidant activities, α-glucosidase inhibitory ability, and sensory evaluation (performed with an electronic nose and tongue and using a panel of 10 assessors)—of tea infusions made from these dried petals. The aim is to furnish valuable insights that can be leveraged for the industrial mass production of premium dried cherry blossoms.

## 2. Materials and Methods

### 2.1. Sample Collection

Cherry blossoms (*Cerasus serrulate ‘Sekiyama’*) at full bloom were harvested from a local farm in Ningbo, China. The collected samples were refrigerated at 4 °C and analyzed within a 3-day period.

### 2.2. Drying Experiments

**Figure 1:**
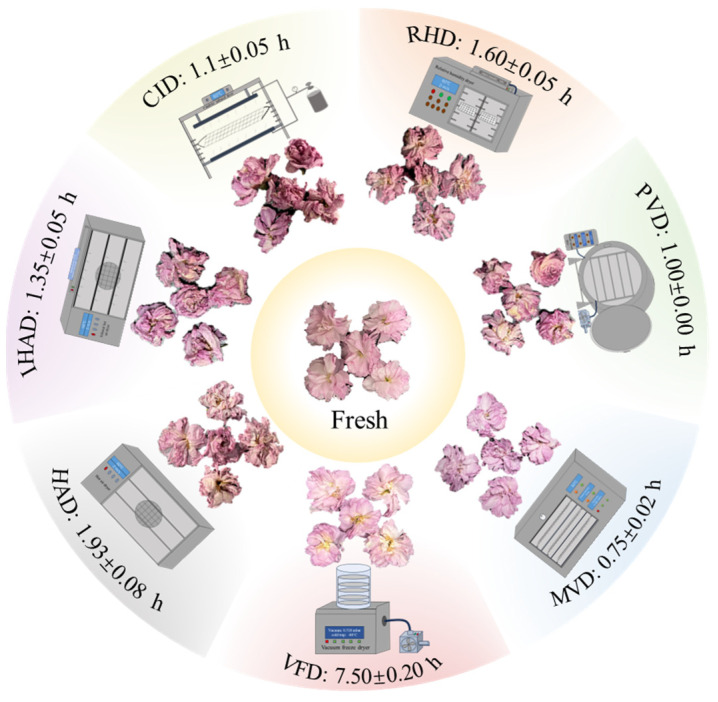
Experimental setup for drying and associated drying times for various techniques—hot-air drying (HAD), infrared hot-air drying (IHAD), catalytic infrared drying (CID), relative humidity drying (RHD), pulsed vacuum drying (PVD), microwave vacuum drying (MVD), and vacuum freeze drying (VFD)—alongside physical illustrations of both fresh and dried cherry blossoms.

To achieve a final dry basis moisture content below 10%, fresh cherry blossoms underwent a series of drying experiments employing various techniques. The drying techniques employed were HAD, IHAD, CID, RHD, PVD, MVD, and VFD. Both fresh and dried petals were analyzed for bioactive compounds, antioxidant activities, and α-glucosidase inhibitory ability and used for preparing tea infusions. The specific protocols for each drying technique are detailed below (Figure 1).

#### 2.2.1. Hot-Air Drying (HAD)

Cherry-blossom petals were uniformly distributed across trays and situated within the drying chamber of a specialized hot-air dryer (DHG-9240A, Shanghai Yiheng Scientific Instrument Co., Ltd., Shanghai, China). The drying parameters were set at an airflow velocity of 2 m/s and a constant temperature of 60 °C.

#### 2.2.2. Infrared Hot-Air Drying (IHAD)

Petals were systematically arranged on trays and introduced into the chamber of an infrared hot-air dryer powered by electricity (self developed by Jiangsu University, Figure 1). The drying parameters included an airflow velocity of 2 m/s, a chamber temperature of 60 °C, and infrared power set at 675 W, as well as a wavelength range of 2.8–3.1 μm. The spatial distance between the sample and the infrared power generator was maintained at 20 cm.

#### 2.2.3. Catalytic Infrared Drying (CID)

In the CID method, petals were evenly spread across trays and put inside the drying chamber of a catalytic infrared dryer powered by natural gas (C3060, Zhenjiang Maybo Innovation Co., Ltd., Zhenjiang, China). The drying conditions were tailored to achieve a surface temperature of 60 °C for the petal samples, while maintaining a 20 cm distance between the sample and the infrared generator (200 °C), as well as a wavelength range of 3.3–8.0 μm.

#### 2.2.4. Relative Humidity Drying (RHD)

Cherry-blossom petals were systematically spread across trays and put inside the drying chamber of a relative humidity dryer (self developed by Jiangsu University, Figure 1). The drying parameters were set at 60 °C, 2 m/s, and a relative humidity level of 30%.

#### 2.2.5. Pulsed Vacuum Drying (PVD)

For PVD, petals were uniformly arranged on trays before being transferred into the drying chamber of a vacuum dryer (DZF-6020, Shanghai Yixin Scientific Instrument Co., Ltd., Shanghai, China). The drying regimen specified a heating plate temperature of 60 °C, with alternating cycles of vacuum (0.518 mbar), and atmospheric pressure applied at 15 min intervals.

#### 2.2.6. Microwave Vacuum Drying (MVD)

In the MVD approach, cherry-blossom petals were uniformly laid out on trays and situated within the chamber of a microwave vacuum dryer (ZK-350A, Qingdao XRTAUTO Co., Ltd., Qingdao, China). Specifically, the predetermined drying parameters encompassed a temperature of 60 °C, a power density of 2 w/g, and a vacuum pressure level of 0.518 mbar.

#### 2.2.7. Vacuum Freeze Drying (VFD)

For VFD, cherry-blossom petals were initially precooled to −40 °C for a duration of 3 h. Subsequently, they were uniformly distributed on trays within the drying chamber of a vacuum freeze dryer (Epsilon 2-6D LSCplus, Martin Christ Ltd., Osterode am Harz, Germany). The tailored drying conditions included a chamber temperature of −18 °C, a vacuum pressure level of 0.518 mbar, and a cold trap temperature set at −80°C.

### 2.3. Preparation of Tea Infusions

The tea-brewing procedure was adapted from Yu et al. [17]. Fresh and dried petals (4 g, dry basis) were immersed in 220 mL of distilled water, preheated to 90 °C, and allowed to steep for a duration of 6 min. Following brewing, the tea infusion was collected and cooled to 25 °C for subsequent analyses, including the measurement of bioactive compounds, antioxidant activities, and α-glucosidase inhibitory ability, as well as sensory properties.

### 2.4. Determination of the Bioactive Compounds

Extraction procedure: fresh and dried petal samples (1.0 g, dry basis) were combined with 10 mL of an 80% methanol solution. Ultrasonic-assisted extraction was subsequently employed, utilizing a power density of 50 W/L and a frequency of 40 kHz. The extraction was carried out for a period of 10 min at an ambient temperature of 25 °C. Following extraction, the mixture was subjected to centrifugation at a rotational speed of 10,000 rpm and a temperature setting of 4 °C for 5 min. The resultant supernatant was collected for subsequent analyses, including the quantification of total phenolic, total flavonoid, and total anthocyanin content. Additionally, evaluations were performed to assess antioxidant activities as well as α-glucosidase inhibitory potential. 

#### 2.4.1. Total Phenolic Content (TPC)

The TPC of fresh petals, dried petals, and tea infusions was quantified employing the Folin–Ciocalteu assay [18]. An aliquot of 0.5 mL from the extracted supernatant (Section 2.4) or tea infusions (Section 2.3) was combined with 2.5 mL of Folin–Ciocalteu reagent at a 5% (*v*/*v*) concentration. This mixture was incubated in darkness for 2 min. Subsequently, 2 mL of a 7.5% (*w*/*v*) Na_2_CO_3_ solution were added, and the mixture was further incubated for 5 min at 50 °C. Absorbance measurements were obtained at 760 nm. The results were expressed as gallic acid equivalents (mg/g, GAE).

#### 2.4.2. Total Flavonoid Content (TFC)

The TFC of fresh petals, dried petals, and tea infusions was quantified with an aluminum chloride colorimetric assay, in accordance with the protocol outlined by Mounir et al. [19]. A 0.5 mL aliquot of the extracted supernatant (Section 2.4) or tea infusions (Section 2.3) was diluted with methanol to a final volume of 1 mL and subsequently mixed with 4 mL of distilled water. To this mixture, 0.5 mL of a 5% sodium nitrite solution were added and incubated for 5 min. Following this, 0.5 mL of 10% AlCl_3_ were introduced, and, then, the solution was left to stand for 6 min. Upon adding 2 mL of 1 M sodium hydroxide, the final volume was adjusted to 10 mL using distilled water. The solution was left to stand for 15 min before absorbance was measured at 510 nm. The results were expressed as quercetin equivalents (mg/g, QE).

#### 2.4.3. Total Anthocyanin Content (TAC)

The pH differential method was employed to assess the TAC of the samples (fresh petals, dried petals, and tea infusions), following the guidelines provided by Xu et al. [20]. A 0.5 mL aliquot of the extracted supernatant (Section 2.4) or tea infusions (Section 2.3) was mixed with 2 mL of a pH 1.0 potassium chloride buffer solution (0.1 M) or with 2 mL of a pH 4.5 sodium acetate buffer solution (0.5 M). The absorbance was measured at wavelengths of 510 nm and 700 nm. The results were quantified as cyanidin-3-glucoside equivalents (mg/g, C3G).

### 2.5. Antioxidant Activities Assay

The antioxidant activities were quantified based on their IC_50_ values, which represent the concentration of the extract necessary to neutralize 50% of the free radical activity. These IC_50_ values were obtained using a linear regression analysis based on the radical-scavenging percentages relative to the sample concentrations, as detailed in the methodology put forth by Feng et al. [8]. Lower IC_50_ values indicate higher antioxidant activity. In this study, antioxidant activities were characterized utilizing both DPPH (1,1-Diphenyl-2-picrylhydrazyl) and ABTS (2,2′-Azinobis-(3-ethylbenzthiazoline-6-sulphonate)) radical-scavenging assays.

#### 2.5.1. DPPH Assay

Aliquots (0.5 mL) of the supernatant at various concentrations (Section 2.4) or tea infusions (Section 2.3) were combined with 2 mL of a 0.2 mM DPPH solution and incubated in darkness for 30 min at 25 °C. The absorbance of the resulting mixture was recorded at 517 nm. The DPPH radical-scavenging activity was quantified as follows in Equation (1):

(1) $$\mathrm{Radical}\;\mathrm{scavenging}\;\mathrm{activity}=\frac{A_{0}-A}{A_{0}}\times 100\%$$

Here, *A*_0_ represents the absorbance of the control solution, which consists of “DPPH + methanol” and *A* indicates the absorbance value of the sample solution, denoted as “DPPH + sample”.

#### 2.5.2. ABTS Assay

Aliquots (0.5 mL) of the supernatant at differing concentrations (Section 2.4) or tea infusions (Section 2.3) were combined with 4 mL of a 0.1 mM ABTS solution and incubated in darkness for 30 min at 25 °C. The absorbance value of the resulting mixture was 734 nm. The ABTS radical-scavenging activity was calculated using Equation 1, which is identical to the equation used for the DPPH assay.

### 2.6. α-Glucosidase Inhibitory Ability (GIA) Assay

Following the methodology proposed by Ismail et al. [21], the GIA of the samples (fresh petals, dried petals, and tea infusions) was assessed. An aliquot of 40 μL of the supernatant (Section 2.4) or tea infusions (Section 2.3) was combined with 130 μL of potassium phosphate buffer (PBS, 100 mM, pH 7) and 60 μL of the substrate solution (5 mM 4-nitrophenyl α-d-glucopyranoside in PBS). An initial absorbance reading (*T*_1_) was taken at 405 nm. Subsequently, 20 μL of α-glucosidase stock solution (1 unit mL^−1^ in PBS) were added. The mixture was incubated for 10 min at a temperature of 37 °C. A final absorbance reading (*T*_2_) was taken at 405 nm. For the negative control, the sample was replaced by blank PBS, and the same steps were followed. Acarbose served as the positive control. The GIA was quantified using Equation (2):

(2) $$\mathrm{GIA}=\left(1-\frac{A_{sample}\left(T_{2}-T_{1}\right)}{A_{negative\;control}\left(T_{2}-T_{1}\right)}\right)\times 100\%$$

where *A_sample_* represents the absorbance of the sample mixture, and *A_negative_*_*control*_ denotes the absorbance of the negative control mixture.

### 2.7. Flavor and Taste Analysis of Tea Infusion

The volatile components (flavor) of the tea infusions prepared from both fresh and dried petals were analyzed using an electronic nose (PEN3, AIRSENSE, Schwerin, Germany), following the procedures detailed by Feng et al. [22]. Briefly, the tea infusion (5 mL) was introduced into a 10 mL headspace vial and allowed to incubate at 35 °C for 10 min. The electronic nose instrument was configured with a gas-flow rate of 450 mL/min and a detection duration of 180 s. Among the recorded signals, the maximum response from the electronic nose sensor was singled out and plotted into a radar chart.

The taste profile of the tea infusions was assessed using an electronic tongue (SA402B, INSENT, Atsugi, Japan), adhering to the methodology outlined by Wei et al. [23]. Briefly, the sensor was immersed in the tea infusion (50 mL) for 120 s, washed, and then immersed in a reference solution for 120 s. The average output of the sensor was then converted into taste information (sourness, bitterness, astringency, aftertaste, umami, richness, saltiness) using calibration procedures and plotted into a radar chart.

### 2.8. Sensory Evaluation by Fuzzy Mathematics with Assessors

According to ISO 5496:2006 [24], ISO 3972:2011 [25], and ISO 11036:2020 [26], sensory analysis methodologies for selection and training of sensory analysis evaluators, 10 assessors (5 males, 5 females, 20–22 years old) majoring in food science were trained, the contents of which included good sensory discrimination ability, evaluation, and description ability. Following the ISO 8589:2007 standard [27], tests were carried out under controlled sensory laboratory circumstances. Ethical approval was obtained for the sensory research, and all the assessors signed consent forms before tastings. 

The appearance of fresh and dried petals was subjectively evaluated by trained assessors, referencing the tea sensory evaluation standards of China (GB/T 23776-2018 [28]). Tea infusions prepared from dried cherry blossoms were evaluated at 10 min intervals, during which the assessors rinsed their mouths with purified water. The evaluation process was adapted from the fuzzy mathematical sensory evaluation method [29]. The teas were differentiated based on drying techniques and subjected to evaluation. 

For the evaluation, a factor set was established comprising taste, flavor, color, and leaf base of the tea infusions. The assessors rated these factors on a grade set (V), which consisted of four levels: excellent (V_1_), very good (V_2_), good (V_3_), and inferior (V_4_). The evaluation adhered to the tea sensory evaluation standards of China (GB/T 23776-2018). Using personal observations and experiences, the assessors subsequently established a weight set (W). Subsequently, the proportion of votes for each factor in each grade was calculated relative to the total number of assessors. These proportions were then converted into approval ratios. By synthesizing these ratios, fuzzy evaluation matrices R_1_–R_7_ were obtained for samples differentiated by drying techniques HAD, IHAD, CID, RHD, PVD, MVD, and VFD, respectively, as follows:

$$R_{1}=\left[\begin{array}{cccc} 0.2 & 0.3 & 0.4 & 0.1\\ 0.1 & 0.1 & 0.4 & 0.4\\ 0.2 & 0.3 & 0.1 & 0.4\\ 0.3 & 0.2 & 0.2 & 0.3 \end{array}\right]\;R_{2}=\left[\begin{array}{cccc} 0.4 & 0.4 & 0.2 & 0.0\\ 0.3 & 0.3 & 0.3 & 0.1\\ 0.3 & 0.4 & 0.3 & 0.0\\ 0.3 & 0.3 & 0.4 & 0.0 \end{array}\right]$$

$$R_{3}=\left[\begin{array}{cccc} 0.4 & 0.4 & 0.2 & 0.0\\ 0.4 & 0.4 & 0.1 & 0.1\\ 0.5 & 0.1 & 0.3 & 0.1\\ 0.6 & 0.3 & 0.1 & 0.0 \end{array}\right]R_{4}=\left[\begin{array}{cccc} 0.5 & 0.4 & 0.1 & 0.0\\ 0.4 & 0.5 & 0.1 & 0.0\\ 0.6 & 0.2 & 0.2 & 0.0\\ 0.7 & 0.2 & 0.1 & 0.0 \end{array}\right]$$

$$R_{5}=\left[\begin{array}{cccc} 0.6 & 0.4 & 0.0 & 0.0\\ 0.6 & 0.4 & 0.0 & 0.0\\ 0.7 & 0.1 & 0.2 & 0.0\\ 0.8 & 0.2 & 0.0 & 0.0 \end{array}\right]R_{6}=\left[\begin{array}{cccc} 0.7 & 0.3 & 0.0 & 0.0\\ 0.6 & 0.4 & 0.0 & 0.0\\ 0.7 & 0.2 & 0.1 & 0.0\\ 0.8 & 0.2 & 0.0 & 0.0 \end{array}\right]$$

$$R_{7}=\left[\begin{array}{cccc} 0.8 & 0.2 & 0.0 & 0.0\\ 0.7 & 0.3 & 0.0 & 0.0\\ 0.8 & 0.2 & 0.0 & 0.0\\ 0.9 & 0.1 & 0.0 & 0.0 \end{array}\right]$$

The fuzzy evaluation matrices were transformed into a comprehensive evaluation set (*Y*) using the following Equation (3):

(3) Y=W×R

To highlight differences in sensory quality between samples, the comprehensive evaluation set was further processed to convert fuzzy sensory scores using an improved methodology of Feng et al. [30]

### 2.9. Statistical Analysis

One-way analysis of variance and correlation analysis were used to further evaluate the data collected, employing IBM SPSS Statistics 25. The experimental design and subsequent data processing were executed using GraphPad Prism 9 and Origin 2021. A threshold for statistical significance was set at *p* < 0.05.

## 3. Results and Discussion

### 3.1. Impact of Drying Techniques on Drying Time and Appearance of Cherry Blossoms

Drying time serves as a crucial factor that influences the quality of dried products. As depicted in Figure 1, significant variations in drying times were observed across different drying techniques. Specifically, MVD had the shortest drying time of 0.75 h, followed by PVD (1.00 h), CID (1.10 h), IHAD (1.35 h), RHD (1.60 h), HAD (1.93 h), and VFD (7.50 h). Xu et al. [31] reported that microwave vacuum drying yielded the shortest drying time for cabbage compared to both hot-air drying and vacuum-freezing drying, owing to the rapid and intense heat generation facilitated by microwave radiation in a constant vacuum setting. Further, several studies have corroborated that reduced drying times can mitigate quality degradation in food products during the drying procedure [32,33]. Therefore, MVD appears to offer the best prospect for minimizing both energy consumption and quality deterioration.

Morphological characteristics, particularly appearance, significantly influence market value and consumer acceptance. Figure 1 illustrates the appearance of both fresh and dried cherry blossoms obtained through various drying techniques. Darker hues were observed in thermally dried samples (MVD, PVD, CID, IHAD, RHD, and HAD), which can be attributed to browning reactions, such as Maillard reactions, caramelization, and ascorbic acid degradation [8]. Notably, MVD-dried samples closely resembled fresh samples in appearance, likely due to the minimal drying time and the vacuum atmosphere that forestalled browning reactions. In contrast, VFD best preserved the original coloration, exhibiting a vivid pink hue. This is plausible because the low-temperature and vacuum conditions effectively inhibited browning and oxidation reactions [34]. These findings are consistent with other studies that have demonstrated VFD’s efficacy in maintaining the original color of food products [35].

### 3.2. Impact of Drying Techniques on the Bioactive Compounds of Petals

**Figure 2:**
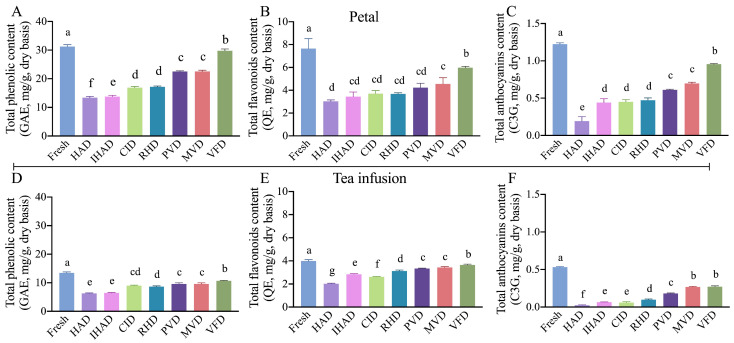
Bioactive compound contents in fresh and dried cherry-blossom products (petal and tea infusion). (**A**–**C**) depict the total phenolic content, total flavonoid content, and total anthocyanin content in fresh and dried petals, respectively. (**D**–**F**) show the same parameters for tea infusions prepared from fresh and dried petals. Different letters denote statistically significant differences (*p* < 0.05) between sample means.

Preserving the quantity and integrity of bioactive compounds in dehydrated plant materials is paramount, given their role in imparting beneficial biological properties for functional and nutraceutical applications [36]. The quantitative analysis of bioactive compounds—namely, TPC, TFC, and TAC—in both fresh and dried petals, is illustrated in Figure 2A–C. Fresh petals demonstrated significantly higher levels (*p* < 0.05) of TPC, TFC, and TAC compared to all the dried samples. This decline can be ascribed to the synergistic effects of elevated thermal exposure and reduced water activity during the drying process [37]. Such reductions in bioactive compounds content (total polyphenol and total flavonoid) postdrying have also been documented in blueberry [38]. 

Significant disparities (*p* < 0.05) in the concentrations of bioactive compounds among petals dried using various techniques were observed in Figure 2A–C. VFD produced the highest retention rates for TPC, TFC, and TAC, at 95.36%, 78.19%, and 78.00%, respectively, which are markedly higher (*p* < 0.05) than those observed in petals dried via thermal techniques, in the following order: MVD, PVD, RHD, CID, IHAD, and HAD. This can be attributed to VFD’s low-temperature and vacuum conditions, which deter thermal degradation and minimize exposure to oxygen, thereby reducing the oxidation and decomposition of bioactive compounds [39]. 

Among the thermally dried petals, those dried using MVD had the highest content of bioactive compounds, second only to petals processed through VFD (a nonthermal technique). This is likely due to MVD’s shorter drying duration and vacuum atmosphere, which minimizes the risk of bioactive compound degradation and enhances the extraction of phenolic compounds [40]. Similarly, Xu et al. [41] found that the TPC and TFC levels in microwave vacuum-dried okra were significantly higher than those in hot-air-dried counterparts and were not significantly different from those in vacuum freeze-dried samples. Conversely, HAD-dried petals exhibited the poorest retention of bioactive compounds, a result attributable to the extended drying period and resultant higher thermal load [8].

### 3.3. Impact of Drying Techniques on Antioxidant Activities of Petals 

**Figure 3:**
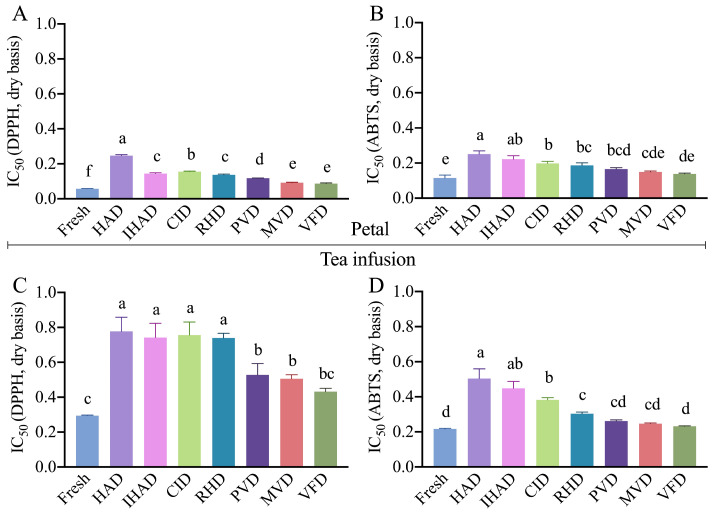
Antioxidant activities in fresh and dried cherry-blossom products (petal and tea infusions). (**A**,**B**) show the IC_50_ values for DPPH and ABTS assays in fresh and dried petals, while (**C**,**D**) display these values for tea infusions prepared from fresh and dried petals. Different letters indicate statistically significant differences (*p* < 0.05) between sample means.

As assessed by DPPH and ABTS assays, the antioxidant activities of petals were differentially influenced by the drying techniques employed. The resulting IC_50_ values are illustrated in Figure 3A,B. Significantly higher IC_50_ values (*p* < 0.05) were observed in the dried petals relative to the fresh samples, indicative of diminished antioxidant activity. This reduction is likely attributable to the degradation of bioactive compounds during the drying process. A similar decline in antioxidant activities has been reported in other foods like tomatoes and ginger after drying [42]. 

**Figure 4:**
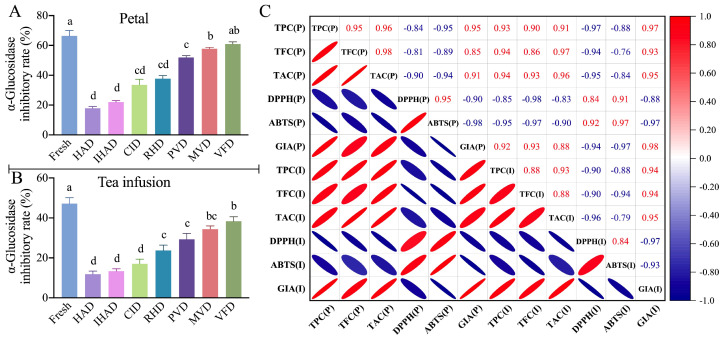
α–Glucosidase inhibitory ability (GIA) in fresh and dried cherry–blossom products (petal and tea infusions). (**A**) illustrates GIA levels in fresh and dried petals, and (**B**) portrays these levels in tea infusions. (**C**) provides a correlation analysis among various physicochemical properties in petals (P) and tea infusions (I), including total phenolic content (TPC), total flavonoid content (TFC), and total anthocyanin content (TAC) and GIA, as well as DPPH and ABTS IC_50_ values. Different letters signify statistically significant differences (*p* < 0.05) between sample means.

The lowest IC_50_ values in both DPPH and ABTS assays demonstrated that VFD was most effective in preserving the antioxidant activity of the dried petals among the tested drying techniques. This was followed, in descending order of effectiveness, by MVD, PVD, RHD, CID, IHAD, and HAD. This trend is congruent with the levels of bioactive compounds present in the dried petals correlation analysis and further substantiated these observations. The IC_50_ values for DPPH and ABTS showed a significantly negative correlation (*p* < 0.05) with the levels of TPC (r = −0.841, −0.946), TFC (r = −0.808, −0.888), and TAC (r = −0.902, −0.944) in the petals, as illustrated in Figure 4C. Similar patterns have been documented in other studies [43]. 

### 3.4. Impact of Drying Techniques on the GIA of Petals

Inhibiting α-glucosidase activity can effectively delay the conversion of carbohydrates to glucose, thereby offering a measure of postprandial blood-glucose control [44]. As displayed in Figure 4A, fresh petals showcased a more potent inhibitory effect on α-glucosidase, with a rate of 66.39%, compared to the range of 17.88–60.98% for dried petals. This suggests that the drying techniques employed led to a decline in the GIA of cherry blossoms. A similar decrease in GIA has been observed in other food items like garlic [45].

In addition, among all drying techniques, petals dried through VFD demonstrated the highest inhibitory rate against α-glucosidase at 60.98%. They were followed by MVD at 57.77%, PVD at 51.88%, RHD at 37.71%, CID at 33.54%, IHAD at 22.03%, and HAD at 17.88%. The variations in these inhibitory rates are likely ascribed to the variations in the concentration of bioactive compounds preserved in the petals. Further supporting these observations, correlation analyses revealed a significant positive association between GIA and the levels of TPC (r = 0.951), TFC (r = 0.854), and TAC (r = 0.907), as depicted in Figure 4C. These findings align with previous studies, which have established that the inhibitory effect on α-glucosidase is correlated with the presence of bioactive compounds like phenolics [46], flavonoids [47], and anthocyanin [48].

In summary, petals dried using VFD were found to be most advantageous in terms of appearance, bioactive compound content, antioxidant activity, and GIA. They largely preserved the quality attributes of fresh cherry blossoms, especially when compared to thermal-drying techniques such as MVD, PVD, RHD, CID, IHAD, and HAD. Among the thermal-drying techniques, MVD not only demonstrated optimal appearance, bioactive compounds, antioxidant capacity, and GIA but also possessed the shortest drying time. To delve deeper into the impact of drying techniques on the quality of cherry blossoms, the quality index of cherry-blossom tea infusions will be analyzed and discussed in subsequent studies. These will encompass a range of factors including bioactive compounds, antioxidant activities, GIA, and sensory properties.

### 3.5. Impact of Drying Techniques on the Quality Attributes of Tea Infusions

#### 3.5.1. Bioactive Compounds, Antioxidant Activities, and GIA

During the brewing process, there was a notable decrease in the content of bioactive compounds (Figure 2D–F), antioxidant activities (Figure 3C,D), and GIA (Figure 4B) in tea infusions compared to both fresh and dried petals. This decline is likely attributable to the elevated temperatures used in brewing, leading to the degradation of bioactive compounds. As both antioxidant activities and GIA are intrinsically linked to the bioactive compounds’ content, they too showed a reduction. Previous research by Abduh et al. [49] indicated that higher brewing temperatures result in decreased levels of bioactive compounds and antioxidant activity in cascara. Similarly, brewing could adversely affect the α-glucosidase inhibitory ability of mulberry petals [50]. 

In terms of tea infusions, those prepared from VFD-dried petals exhibited the highest levels of bioactive compounds, antioxidant activities, and GIA among all tested infusions. This was followed in descending order by MVD, PVD, RHD, CID, IHAD, and HAD. This pattern mirrors the trends observed in the dried petals. Further corroboration for this trend comes from a correlation analysis, which revealed strong positive correlations between the bioactive compound content—namely TPC (r = 0.933), TFC (r = 0.855), and TAC (r = 0.959)—as well as antioxidant activities (DPPH IC_50_ value r = 0.839 and ABTS IC_50_ value r = 0.966), and GIA (r = 0.975) in both the dried petals and the tea infusions (Figure 4C).

#### 3.5.2. Flavor and Taste

Flavor and taste are critical quality metrics for evaluating cherry blossoms as an edible flower. A quantitative analysis of these attributes in tea infusions—prepared both from fresh and dried petals—was performed using an electronic nose and tongue.

**Figure 5:**
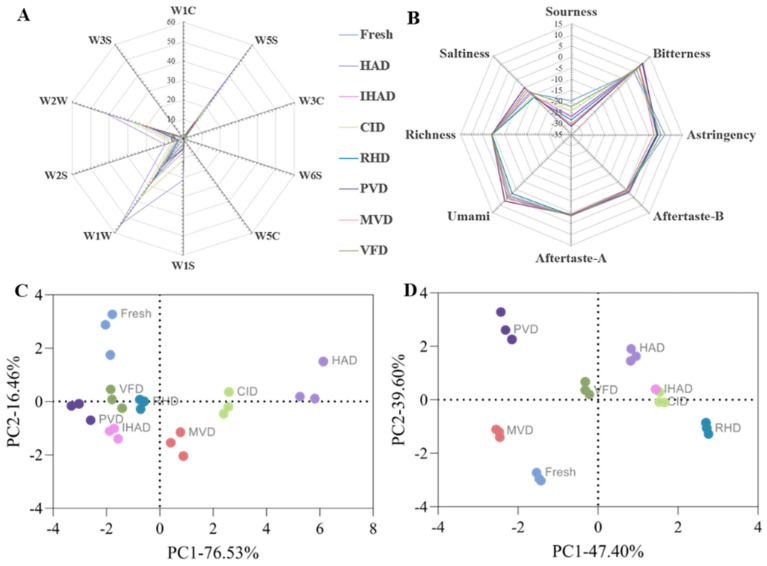
Principal–component analysis (PCA) and radar plots for sensor response values in tea infusions prepared from fresh and dried petals, as captured by electronic nose and electronic tongue. (**A**,**B**) display PCA results for electronic nose and tongue measurements. (**C**) shows the radar plot for sensor response values in tea infusions as detected by the electronic nose, covering a range of analytes, including W1C—aromatic, benzene; W5S—nitrogen oxides; W3C—aromatic, ammonia; W6S—hydrogen; W5C—arom–aliph; W1S—broad–methane; W1W—sulfur–organic; W2S—broad–alcohol; W2W—sulph–chlor; and W3S—methane–aliph. (**D**) presents the radar plot for sensor response values as measured by the electronic tongue.

Flavor profiles were assessed using an electronic nose, with the results represented in a radar plot (Figure 5C). The response values for W2W(sulfur-chlorine), W2S(broad-alcohol), W1W(sulfur-organic), W1S(broad-methane), and W5S (broad range) increased in the tea infusions made from dried petals compared to those prepared from fresh petals. These changes could be attributed to chemical alterations induced by the drying process, such as Maillard reactions, molecular interactions, and the degradation of larger molecules, resulting in the formation of new volatile compounds [8]. The response values for all sensors were highest in the tea infusion prepared with HAD-dried petals, likely due to the extended heat processing, which generated a richer volatile component profile. Dong et al. [51] similarly reported that HAD significantly changed the volatile compound profile in dried coffee beans. Importantly, the tea infusion made from VFD-dried petals most closely resembled the fresh sample, supporting earlier findings that vacuum freeze drying is effective at preserving natural aroma [35].

Taste attributes were examined using an electronic tongue, with results illustrated in a radar plot (Figure 5D). The sourness and astringency of tea infusions made from dried petals were reduced, while bitterness increased compared to infusions made from fresh petals. This trend has also been observed in dried shiitake mushrooms [52]. Additionally, the taste profile of the tea infusion prepared from VFD-dried petals was the most similar to that of the infusion made from fresh petals, corroborating the results for volatile components.

Principal–component analysis (PCA) served as the analytical technique for delineating differences in the flavor and taste profiles across the range of tea infusions (Figure 5A,B). PC1 and PC2 jointly account for 92.99% and 87.00% of the variable contributions in flavor and taste, respectively, which results in an appreciable compression of the data. Table 1 presents the Varimax rotated PC factor loadings, revealing the correlations between the PCs and the original data. Notable associations are evident in loadings exceeding 0.5. The PCA of flavor data revealed that PC1 was significantly associated with multiple flavor characteristics (W1C, W5S, W3C, W6S, W5C, W1S, W1W, W2S, and W2W), excluding W3S, while PC2 displayed a significant correlation with W3S of the tea infusion related to cherry blossom. Moreover, W2W, W1W, W2S, W1S, and W5S are the primary flavor components of PC1, suggesting that these parameters significantly contribute to the flavor profile of tea infusions. Consequently, variations in these parameters resulting from drying techniques can significantly influence the overall flavor of the tea infusions. In terms of taste, PC1 is primarily linked to sourness, aftertaste-A, umami, and saltiness, while PC2 is associated with bitterness, astringency, aftertaste-B, aftertaste-A, and richness. The primary taste component of PC1 is sourness, indicating that the drying technique substantially impacts the sourness of the tea infusion and significantly influences the taste of the tea infusions.

The PCA results indicate that the sample points of tea infusions prepared with the same drying method are closer in distance, signifying a higher similarity within tea infusions prepared using the same drying method. The infusions prepared from fresh petals are distinctly separated from those made with dried petals, indicating that drying techniques significantly influence the infusion’s flavor and taste. In summary, preserving the original flavor and taste is of paramount importance for cherry-blossom tea infusions. Based on the observed results, VFD provides a feasible method for achieving this goal.

#### 3.5.3. Fuzzy Mathematical Sensory Evaluation

Sensory evaluation is pivotal for gauging consumer acceptance of new food products and for understanding preferences in various culinary contexts [53]. In this experiment, the weights for different sensory attributes were allocated as follows: W = (35% of flavor, 30% of taste, 15% of color, and 20% of leaf base). Flavor, thus, has the highest weight among the four evaluation criteria, underscoring its critical role in determining the quality of cherry-blossom tea infusions. 

The fuzzy logic approach, known for its ease of implementation, flexibility, and tolerance for imprecise data, is particularly suitable for modeling the intricate, nonlinear relationships inherent in sensory studies [54]. Comprehensive sensory evaluation scores for each sample are presented in Table 2. The tea infusion prepared from VFD-dried petals received the highest overall score, which can likely be attributed to VFD’s efficacy in preserving both aroma and taste. Consistent with this, vacuum freeze drying has been shown to successfully maintain the aroma and structural integrity of roses, yielding rose-tea preparations that outperform those prepared using alternative drying techniques, such as hot-pump drying, hot-air drying, relative humidity drying, and catalytic infrared drying [30]. Tea infusions made from thermally dried petals generally received lower scores, with the HAD method performing particularly poorly. This diminished quality may stem from the introduction of new flavor and taste compounds as well as the deterioration in appearance, which failed to meet the aesthetic expectations of the evaluators. Taken together, these findings imply that the quality of dried cherry-blossom tea infusions can vary significantly depending on the chosen drying technique, reinforcing the need for judicious selection of an optimal method.

## 4. Conclusions

This study comprehensively evaluates the impacts of various drying techniques—including HAD, IHAD, CID, RHD, PVD, MVD, and VFD—on multiple attributes of cherry-blossom products, including drying time, appearance, bioactive compound content, antioxidant activities, GIA, flavor, taste, and sensory evaluation, both in petal form and as tea infusions. Among these techniques, VFD-dried petals exhibited superior appearance, bioactive compounds, antioxidant activities, and GIA. However, the VFD technique also demonstrated the lowest efficiency in terms of drying time. Postbrewing, tea infusions prepared from these dried petals largely retained the quality attributes of the initial dried material. Specifically, VFD-dried petals yielded tea infusions with flavor and taste profiles most closely resembling those of infusions prepared from fresh petals and received the highest sensory evaluation scores. In the realm of thermal drying techniques—comprising HAD, IHAD, CID, RHD, PVD, and MVD—MVD emerged as the most promising, second only to VFD. It not only excelled in preserving bioactive compounds, antioxidant activities, GIA, and sensory among the thermal techniques, but also achieved the shortest drying time. Consequently, for the production of high-quality dried cherry-blossom products, VFD stands out as the method of choice. However, when considering a balance between efficiency and product quality, MVD presents itself as the most viable alternative to VFD.

## Figures and Tables

**Table 1:** Varimax rotated PC factor loadings for the specific parameters of the flavor and taste of cherry-blossom tea infusions.

Var (Flavor)	PC1	PC2	Var (Taste)	PC1	PC2
W1C	−0.792	0.491	Sourness	0.954	−0.208
W5S	0.953	0.148	Bitterness	−0.063	0.684
W3C	−0.836	0.506	Astringency	0.421	0.799
W6S	0.832	0.439	Aftertaste-B	0.299	0.764
W5C	−0.797	0.436	Aftertaste-A	0.615	0.633
W1S	0.964	0.201	Umami	−0.978	0.175
W1W	0.989	−0.045	Richness	−0.333	0.765
W2S	0.989	0.066	Saltiness	−0.919	0.312
W2W	0.994	0.050			
W3S	0.467	0.834			

**Table 2:** Comprehensive sensory evaluation results of cherry-blossom tea infusions prepared by different drying techniques.

Drying Techniques	Y_n_ Comprehensive Evaluation Set	Comprehensive Score
HAD	Y_1_ = {0.190, 0.220, 0.315, 0.275}	2.325
IHAD	Y_2_ = {0.335, 0.350, 0.285, 0.030}	2.990
CID	Y_3_ = {0.445, 0.335, 0.165, 0.045}	3.160
RHD	Y_4_ = {0.525, 0.360, 0.115, 0.000}	3.410
PVD	Y_5_ = {0.665, 0.315, 0.030, 0.000}	3.665
MVD	Y_6_ = {0.690, 0.295, 0.015, 0.000}	3.675
VFD	Y_7_ = {0.790, 0.210, 0.000, 0.000}	3.790

## Data Availability

The data presented in this study are available on request from the corresponding author. The data are not publicly available as it is contained within the article.
